# Marked genetic diversity within *Blastocystis* in Australian wildlife revealed using a next generation sequencing–phylogenetic approach

**DOI:** 10.1016/j.ijppaw.2023.100902

**Published:** 2023-12-28

**Authors:** Anson V. Koehler, H.M.P. Dilrukshi Herath, Ross S. Hall, Stephen Wilcox, Robin B. Gasser

**Affiliations:** a Department of Veterinary Biosciences, Faculty of Science, The University of Melbourne, Parkville, Victoria, 3010, Australia; b Walter and Eliza Hall Institute, Parkville, Victoria, 3052, Australia

**Keywords:** Blastocystis, Subtypes (STs), Wildlife, Marsupials, Deer, Next generation sequencing (NGS), Targeted amplicon sequencing

## Abstract

*Blastocystis* is a genus of intestinal stramenopiles that infect vertebrates, and may cause disease of the alimentary tract. Currently, at least 40 genotypes (“subtypes”) of *Blastocystis* are recognised worldwide based on sequence data for the small subunit of the nuclear ribosomal RNA (*SSU-rRNA*) gene. Despite the numerous studies of *Blastocystis* worldwide, very few studies have explored *Blastocystis* in wild animals, particularly in Australia. Here, we used a PCR-based next generation sequencing (NGS)–phylogenetic approach to genetically characterise and classify *Blastocystis* variants from selected wildlife in the Australian state of Victoria. In total, 1658 faecal samples were collected from nine host species, including eastern grey kangaroo, swamp wallaby, common wombat, deer, European rabbit, canines and emu. Genomic DNA was extracted from these samples, a 500 bp region of the *SSU-rRNA* gene amplified by polymerase chain reaction (PCR) and, then, a subset of samples sequenced using Illumina technology. Primary PCR detected *Blastocystis* in 482 of the 1658 samples (29%), with the highest percentage in fallow deer (63%). Subsequent, Illumina-based sequencing of a subset of 356 samples revealed 55 distinct amplicon sequence variants (ASVs) representing seven currently-recognised subtypes (STs) [ST13 (prominent in marsupials), ST10, ST14, ST21, ST23, ST24 and ST25 (prominent in deer)] and two novel STs (ST45 and ST46) in marsupials. Mixed infections of different STs were observed in macropods, deer, emu and canids (fox, feral dog or dingo), but no infection was detected in rabbits or wombats. This study reveals marked genetic diversity within *Blastocystis* in a small number of species of wild animals in Australia, suggesting complexity in the genetic composition and transmission patterns of members of the genus *Blastocystis* in this country.

## Highlights

• *Blastocystis* was detected in Australian wildlife, including macropods and deer, using relatively large sample sizes
• Two novel subtypes ST45 and ST46 were detected in marsupials
• Mixed subtypes were observed in macropods, deer, emu and canids, but no infection was detected in rabbits or wombats
• Occurrence of human-infective subtypes in wildlife present is a potential risk for the contamination of water catchments

## Graphical abstract

**Figure:**
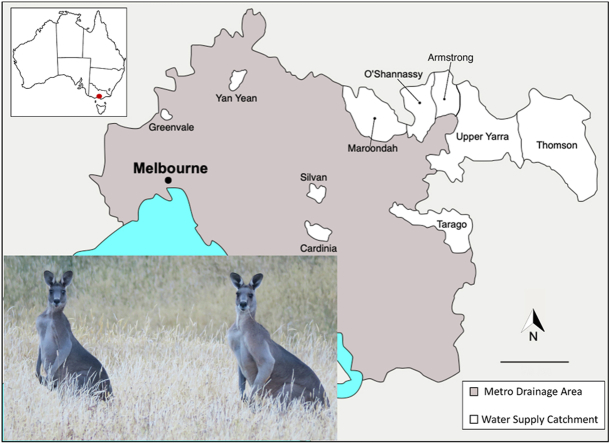

## 1 Introduction

*Blastocystis* is a stramenopile, occurring in the gastrointestinal tracts of humans and a range of other animals worldwide (Hublin et al., 2021). *Blastocystis* infection can be associated with gastrointestinal symptoms, including diarrhoea, bloating, vomiting, abdominal pain, nausea and/or urticaria, although the pathogenicity of this group has been somewhat controversial due to asymptomatic infections in healthy individuals (Stensvold et al., 2009b; Roberts et al., 2013; Andersen and Stensvold 2016; Deng et al., 2021).

Previous studies have reported the occurrence of *Blastocystis* in a range of domestic and wild animal hosts in various countries around the world (reviewed by Stensvold and Clark, 2016; Hublin et al., 2021). Given that most *Blastocystis* subtypes (STs) are not host specific, zoonotic transmission, between animals and humans is likely. This is evidenced by reports of the transmission of some *Blastocystis* STs (i.e., ST5 and ST6) from pigs and chickens to humans (Wang et al., 2014; Greige et al., 2018). Thus, studying the occurrence, prevalence and diversity of *Blastocystis* STs in both wild and domestic animal populations is important to understand the epidemiology of blastocystosis.

In Australia, most published studies were focused on animals in zoos, farms and veterinary practices using relatively small sample sizes (e.g., Parkar et al., 2010; Roberts et al., 2013; Wang et al., 2014), and there is very limited information for wildlife in this country (Roberts et al., 2013; Parkar, 2016). Therefore, there is a need to expand genetic explorations of *Blastocystis* in wildlife to gain a better appreciation of the nature and extent of genetic variability within this genus in Australia, and to identify potential reservoirs for transmission to humans.

Globally, there is considerable genetic diversity within *Blastocystis* based on PCR-based analyses of small subunit ribosomal RNA (*SSU-rRNA*) gene, with 41 STs reported to date. Currently, 40 of these STs (i.e., ST1-ST17, ST21 and ST23-ST44) are recognised as legitimate (Maloney et al., 2020, 2021; Santín et al., 2023), 14 of which (ST1-ST10, ST12, ST14, ST16 and ST23) are classified as ‘zoonotic’ because they have been recorded in both humans and other animals (including pigs, chickens and non-human primates) (Stensvold and Clark 2016; Maloney et al., 2023). PCR-based sequencing has been useful for detecting and defining *Blastocystis* STs in many different host species (Scicluna et al., 2006; Santín et al., 2011), and next generation sequencing (NGS) shows utility to identify and characterise multi-ST infections in individual animals with high sensitivity and specificity (Maloney and Santín 2021). In the present study, we utilised a PCR-based NGS approach to assess genetic diversity within *Blastocystis* from at least nine species of animals, representing marsupials, deer, lagomorphs, canids and birds, living in drinking water catchment areas in Melbourne, Victoria, Australia, in all four seasons from 2009 to 2022.

## 2 Material and methods

### 2.1 Samples

**Fig. 1:**
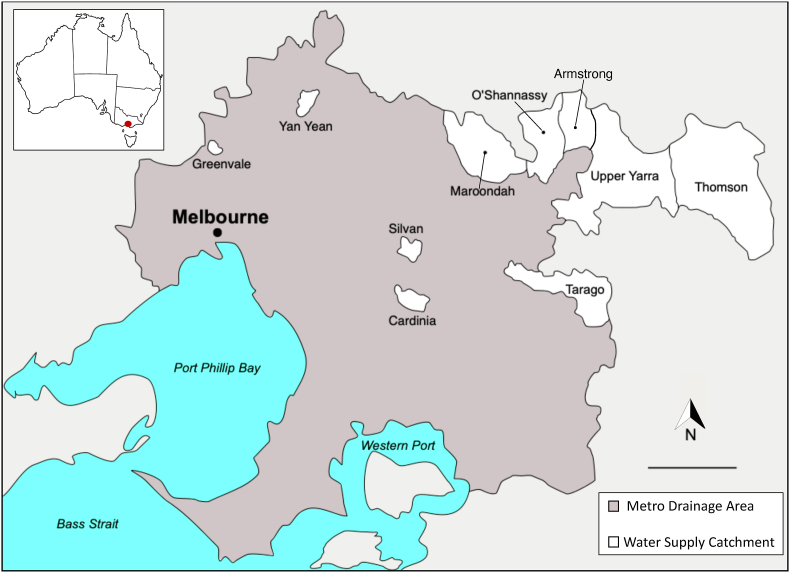
Map showing Melbourne's water catchment areas where samples were collected (2009-2022).

Between 2009 and 2022, 1658 faecal samples were collected from eastern grey kangaroo (n = 636), swamp wallaby (106), common wombat (94), red deer (70), fallow deer 49), sambar deer (398), deer (species not identified; 23), European rabbit (94), emu (94) and canid (fox, feral dog or dingo; 94) for subsequent DNA extraction (Table 1, Supplementary file 1). Fresh scats were collected from the ground from Melbourne's water catchment areas: Armstrong (37°38′S, 145°51′E), Yan Yean (37°33′S, 145°08′E), Greenvale (37°37′S, 144°54′E), Upper Yarra (37°40′S, 145°55′E), O'Shannassy (37°40′S, 145°48′E), Maroondah (37°38′S, 145°33′E), Silvan (37°50′S, 145°25′E) and Cardinia (37°47′S, 145°24′E) in Victoria, Australia (Fig. 1). Host species were identified based on the morphological (Triggs, 2004) and/or molecular (Koehler et al., 2020) identification of scats.

**Table 1:** Summary of the occurrence and diversity of *Blastocystis* sequence types (STs) in animal species inhabiting Melbourne's water catchments (2009-2022); *Blastocystis* STs observed in marsupials, deer and other host species in the initial PCR analysis, and the STs and percentage of each subtype observed in each animal species through the analysis of NGS-derived data.

Host (Scientific name)	Initial PCR			Illumina sequencing									
	No. of samples tested	No. of positives (%)		No. samples in NGS analysis	Number (percentage) of each subtype observed								
					ST46	ST45	ST10	ST13	ST14	ST21	ST23	ST24	ST25
***Macropods***													
Eastern grey kangaroo (*Macropus giganteus*)	636	221 (34.8)		180	169 (93.9)	2 (1.1)	0 (0.0)	168 (93.3)	2 (1.1)	2 (1.1)	0 (0.0)	1 (0.6)	0 (0.0)
Swamp wallaby (*Wallabia bicolor*)	106	12 (11.3)		6	2 (33.3)	0 (0.0)	1 (16.7)	6 (100.0)	0 (0.0)	0 (0.0)	0 (0.0)	0 (0.0)	0 (0.0)
**Total - macropods**	**742**	**233**		**186**	**171 (91.9)**	**2 (1.0)**	**1 (0.5)**	**174 (93.5)**	**2 (1.0)**	**2 (1.0)**	**0 (0.0)**	**1 (0.5)**	**0 (0.0)**
***Deer***													
Red deer (*Cervus elaphus*)	70	19 (27.1)		10	0 (0.0)	0 (0.0)	6 (60.0)	0 (0.0)	8 (80.0)	9 (90.0)	4 (40.0)	10 (100.0)	3 (30.0)
Fallow deer (*Dama dama*)	49	31 (63.3)		25	0 (0.0)	0 (0.0)	22 (88.0)	0 (0.0)	24 (96.0)	22 (88.0)	18 (72.0)	25 (100.0)	19 (76.0)
Sambar deer (*Rusa unicolor*)	398	192 (48.2)		132	0 (0.0)	0 (0.0)	102 (77.3)	1 (0.8)	93 (70.5)	125 (94.7)	90 (68.2)	120 (90.9)	96 (72.7)
Unknown deer	23	4 (17.4)		0	0 (0.0)	0 (0.0)	0 (0.0)	0 (0.0)	0 (0.0)	0 (0.0)	0 (0.0)	0 (0.0)	0 (0.0)
**Total - deer**	**540**	**246**		**167**	**0 (0.0)**	**0 (0.0)**	**130 (77.8)**	**1 (0.6)**	**125 (74.8)**	**156 (93.4)**	**112 (67)**	**155 (92.8)**	**118 (70.7)**
***Other***													
Emu (*Dromaius novaehollandiae*)	94	2 (2.1)		2	1 (50.0)	0 (0.0)	0 (0.0)	1 (50.0)	1 (50.0)	1 (50.0)	1 (50.0)	1 (50.0)	1 (50.0)
Canid	94	1 (1.1)		1	1 (100.0)	0 (0.0)	0 (0.0)	1 (100.0)	0 (0.0)	0 (0.0)	0 (0.0)	0 (0.0)	0 (0.0)
Common wombat (*Vombatus ursinus*)	94	0 (0)		0	0 (0.0)	0 (0.0)	0 (0.0)	0 (0.0)	0 (0.0)	0 (0.0)	0 (0.0)	0 (0.0)	0 (0.0)
European rabbit (*Oryctolagus cuniculus*)	94	0 (0)		0	0 (0.0)	0 (0.0)	0 (0.0)	0 (0.0)	0 (0.0)	0 (0.0)	0 (0.0)	0 (0.0)	0 (0.0)
**Total - other**	**376**	**3**		**3**	**2 (66.7)**	**0 (0.0)**	**0 (0.0)**	**2 (66.7)**	**1 (33.3)**	**1 (33.3)**	**1 (33.3)**	**1 (33.3)**	**1 (33.3)**
**Total**	**1658**	**482**		**356**	**173**	**2**	**131**	**177**	**128**	**159**	**113**	**157**	**119**

### 2.2 DNA extraction and PCR

Genomic DNA was isolated from individual faecal samples (∼0.25 g of faeces, taken from the centre of pellets) using the DNeasy PowerSoil Pro kit (Qiagen, Netherlands), according to manufacturer's instructions, and stored at −80 °C until use. A short region of *SSU*-*rRNA* (∼500 bp; cf. Santín et al., 2011) was amplified (i.e., PCR-1) from individual faecal DNA samples using the primers Blast505_532mod (forward: 5′-GTG ACC TAT GAA CTC AGG AGT CGG AGG TAG TGA CAA TAA ATC-3′) and Blast998_1017mod (reverse: 5′-CTG AGA CTT GCA CAT CGC AGC TGC TTT CGC ACT TGT TCA TC-3′) (Santín et al., 2011). Each PCR was conducted in a volume of 25 μl containing 0.2 μM of each the forward and reverse primer, 0.31 U of Taq enzyme mix (2x; New England Biolabs, Ipswich, MA, USA) and 20-50 ng of DNA template (except for the negative controls, which contained no DNA). The cycling conditions were: an initial denaturation of 95 °C for 3 min, followed by 30 cycles of 95 °C for 15 s (denaturation), 55 °C for 30 s (annealing), 72 °C for 30 s (extension), followed by final extension of 72 °C for 7 min. PCR products were examined following electrophoresis in 1.5% agarose gels in 65 mM Tris-HCl, 27 mM boric acid, 1 mM EDTA, pH 9 - TBE (Bio-Rad, Hercules, CA, USA) and staining in ethidium bromide. A 100 bp ladder (Promega, Madison, WI, USA) was used as a size marker. A set of known positive-control and *Blastocystis-*positive samples were selected (see Supplementary file 1) and used in all PCR and sequencing runs.

### 2.3 PCR-based NGS, data processing and phylogenetic analysis

NGS was conducted using an established method (cf. Aubrey et al., 2015). In brief, amplicons from the primary PCR (i.e., PCR-1, see subsection 2.2) were purified using 1X Ampure beads (Beckman Coulter, Brea, CA, USA). Secondary PCR (PCR-2) was performed to introduce the indexing primers to the 5′-end of the products, allowing the multiplexing of amplicons for subsequent sequencing using Illumina technology. In total, 384 samples (including a range of well-defined positive and negative controls) were randomised amongst four plates and multiplexed using 16 forward and 24 reverse indices. Cycling conditions for PCR-2 were: initial denaturation of 95 °C for 3 min, 15 cycles of 95 °C for 15 s (denaturation), 60 °C for 30 s (annealing) and 72 °C for 30 s (extension), followed by a final extension of 72 °C for 7 min. Following PCR-2, five randomly-selected, multiplexed amplicons from each of the four plates were examined for quality using an Agilent 2200 TapeStation (Agilent, Santa Clara, CA, USA). The individual multiplexed-amplicons from each plate were combined into four separate pools, purified using 0.7X Ampure Beads to remove the primer dimers and assessed for quality using the TapeStation. A final pool was prepared by combining all four pools, DNA was quantified using a Qubit 2.0 Fluorometer (Life Technologies, Carlsbad, CA, USA), and then sequenced on an Illumina MiSeq 600-cycle using v3 chemistry (Illumina, San Diego, CA, USA) at the Walter and Eliza Hall Institute of Medical Research (WEHI), Victoria, Australia.

Sequence data obtained were de-multiplexed using in-house software. Gene-specific primers, overhang primers and index sequences were removed using Cutadapt (Martin, 2011), and reads were imported into R v4.1.0 (R Core Team, 2021). Subsequently, reads were filtered for quality and length, de-replicated and chimeras removed, and only high-quality reads were retained. Amplicon sequence variants (ASVs) were defined using a DADA2 workflow (Callahan et al., 2016; Lee, 2019), whereby initial taxonomy was assigned using IDTAXA (Murali et al., 2018), employing a curated reference library of known *Blastocystis* STs (http://entamoeba.lshtm.ac.uk/ref.blasto.txt, accessed on 5 March 2023). ASVs represented by > 100 sequence reads were retained; chimeras identified were excluded; and ASV taxonomies were established based on the best homology match to sequences in a curated reference library (http://entamoeba.lshtm.ac.uk/ref.blasto.txt), and a BLASTn search (https://blast.ncbi.nlm.nih.gov), followed by a phylogenetic analysis of ASVs and top BLAST matches. The phylogenetic tree (Supplementary file 2) was generated using neighbor-joining method in MEGA 11 (Tamura et al., 2021) from a trimmed MAFFT alignment. All fastq files from this study are available in NCBI under BioProject number PRJNA987751. The sequence data obtained were displayed and examined using the R package “Animalcules” (Zhao et al., 2021).

### 2.4 PCR-based sequencing of a long SSU-rRNA gene region and phylogenetic analysis of sequence data to confirm recognised and new STs

**Fig. 2:**
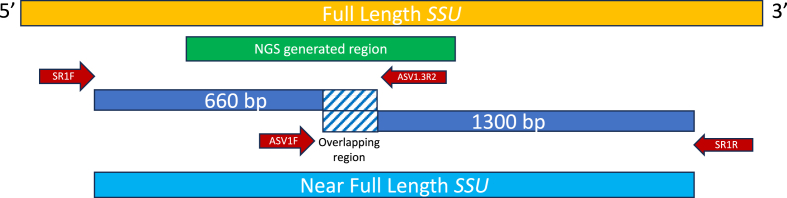
Diagram of the method used to obtain sequence for a *SSU*-rRNA gene region (∼1750 bp) of *Blastocystis*. Two primer sets were used to obtain overlapping sequences for this region.

Following phylogenetic analysis (sub-section 2.3), selected ASVs, including those representing novel STs, were verified by PCR-based sequencing of two overlapping regions (660 and 1300 bp; Fig. 2) of *SSU-rRNA* (length: 1717 to 1765 bp) using primer pairs listed in Table 2. Samples identified to represent one ASV were selected, and sequence chromatograms obtained were scrutinised to exclude sequence heterogeneity/polymorphism. This approach allowed the verification of sequence overlap and exclusion of artefacts (including chimeras).

**Table 2:** The primers used to obtain long *SSU-rRNA* gene sequences (∼1750 bp) for selected, recognised subtypes (STs) and the two novel STs (ST45 and ST46) identified in the present study. All primers starting with “ASV” were designed specifically for this study.

ASV	Forward primer	Reverse primer	Product size (bp)	Annealing conditions	Sample(s) Tested
ASV1	SR1F^a^ - 5′ GCTTATCTGGTTGATCCTGCCAGT 3′	ASV1.3R2 - 5′AAATGACCAACCATTACTACAAG 3′	660	56 °C for 30s	C11807, MR11989
	ASV1F - 5′ GAAGTGTGGGGCAAACTATTAT 3′	SR1R^a^ - 5′ TTGATCCTTCCGCAGGTTCACCTA 3′	1300	56 °C for 60s	
ASV3	SR1F^a^	ASV1.3R2 - 5′AAATGACCAACCATTACTACAAG 3′	660	56 °C for 30s	SV11234
	ASV1F - 5′ GAAGTGTGGGGCAAACTATTAT 3′	SR1R^a^	1300	56 °C for 60s	
ASV4	SR1F^a^	ASV4R - 5′ GAGAGGGGGTGAGCAAATAA 3′	660	56 °C for 30s	C9928
	ASV4F - 5′ TTGAAGTGTGGGGTGAAAAA 3′	SR1R^a^	1300	56 °C for 60s	
ASV6	SR1F^a^	ASV6R2 - 5′GACCAACTCCCATGTACAGA 3′	660	56 °C for 30s	MR10657
	ASV6F - 5′ GAAGTGAAGGTGTTTGTATATTG 3′	SR1R^a^	1300	56 °C for 60s	
ASV11	SR1F^a^	ASV11.2R - 5′ GACATTGGAATGAGGAATTTAGA 3′	660	56 °C for 30s	SV7134, SV10980
	ASV11.2F - 5′ GGAATTTGTTTATTATATGGCTTTG 3′	SR1R^a^	1300	56 °C for 60s	
ASV28	SR1F^a^	ASV28R - 5′ GAAATGATCAATGGGAAAGGAA 3′	660	56 °C for 30s	GV4429
	ASV28F - 5′ AATGTGTTAGGGGTCGGTTTC 3′	SR1R^a^	1300	56 °C for 60s	
	NB1F^b^ - 5′ GTGATGGGGATTGATGCTTG 3′	SR1R^a^	270	58 °C for 30s	

a Yoshikawa et al. (2000).

b Stensvold et al. (2011)

PCR was conducted in 50 μl containing 25 mM of MgCl_2_ (Promega, Madison, USA), PCR buffer (5x; Promega), 10 mM of dNTPs (Promega), 100 pmol of each forward and reverse primers to the *SSU*-*rRNA* gene, 1.25 U of Go*Taq* DNA polymerase and 20-50 ng of DNA template, except in negative controls. The cycling conditions were: initial denaturation of 94 °C for 5 min, followed by 35 cycles of 94 °C for 60 s (denaturation), 56 °C - 58 °C for 30 - 60 s (depending on the sample tested; annealing), 72 °C for 90 s (extension), and the final extension of 72 °C for 10 min (Table 2). The PCR products were visualised on 1.5% agarose gels using ethidium bromide, and using 100 bp ladder (Promega, Madison, WI, USA) as a size marker. Amplicons were treated with shrimp alkaline phosphatase and exonuclease I (ThermoFisher, Waltham, USA) according to manufacturer's instructions, and then directly sequenced using the Sanger method (Crossley et al., 2020). Sequences were assembled using Geneious Prime software v2023.0.4; these sequences were independently verified by PCR-based sequencing using multiple primers (Table 2) positioned within the *SSU*-*rRNA* gene. All sequences are available from GenBank under accession numbers OR192515 to OR192520. Pairwise distances between sequences were calculated using Geneious Prime v. 2023.0.4.

Subsequently, a phylogenetic tree was constructed using *SSU-rRNA* sequence data, employing selected sequences obtained from the reference data base (http://entamoeba.lshtm.ac.uk/blastorefseqs.htm; accessed on 5 March 2023) and other sequences representing *Blastocystis*, available via GenBank, employing *Proteromonas lacertae* as an outgroup. Sequences were aligned using MUSCLE (Edgar, 2004), followed by manual adjustment using Mesquite v.3.61 (Maddison and MaddisonMesquite, 2015). Phylogenetic analyses of the data were conducted by Bayesian inference (BI) using MrBayes v.3.2.6 (Ronquist et al., 2012), with the likelihood parameters based on the Akaike Information Criteria test in IQ-TREE v.2 (Minh et al., 2020). Specifically, the number of substitutions (Nst) was set at six, and an invariant gamma-distribution was used. Posterior probability (pp) values were calculated from 2,000,000 generations using four simultaneous tree-building chains, with trees being saved every 100th generation. The standard deviation of split frequencies was <0.01, and the potential scale reduction factor approached one, indicating convergence. To ensure convergence and insensitivity to priors, the analyses were run three times. Finally, a 50% majority rule consensus tree was constructed based on the final 75% of trees generated by BI. Novel STs were designated using the guidelines by Stensvold and Clark (2020).

### 2.5 Statistical analysis

Statistical analysis was performed to assess the seasonal effect on the occurrence of *Blastocystis* in macropods (kangaroo and wallaby) and deer (red, fallow and sambar deer) using R. The binary logistic regression method was used, with season as the predictor variable, and the presence/absence of the *Blastocystis* was used as the output variable; the odds ratio (OR) and 95% confidence interval (CI) were calculated. Chi-square (ᵡ^2^) was also calculated using Binomial ANOVA method. The *p* value of <0.05 was considered as statistically significant.

## 3 Results

### 3.1 Amplicons, and NGS data sets produced and curated

From the 1658 faecal DNA samples available, 482 primary *SSU*-*rRNA* amplicons (500 bp) representing *Blastocystis* were produced (overall prevalence: 29.1%) (Table 1, Supplementary file 1). Most amplicons represented fallow deer (63.3% of 49), followed by sambar deer (48.2% of 398), kangaroo (34.8% of 636), red deer (27.1% of 70), wallaby (11.3% of 106), emu (2.1% of 94) and feral dogs (1.1% of 94). No amplicons were produced from the 94 samples from rabbits or the 94 samples from the common wombat (see Table 1).

A total of 4,603,282 sequence reads were produced by NGS from all 384 samples, and 3,882,168 reads were retained following initial filtering. Following dereplication, chimera detection and filtering (using a cut-off of ≤5), 2,263,566 high-quality forward-reads, with an average of 6358 reads per sample, were obtained. Reverse reads failed quality control and were not used. Three of four negative controls (one in each plate) were free from reads, although one of these controls contained 43 reads.

### 3.2 Nine Blastocystis STs defined by NGS and phylogenetic analysis

**Fig. 3:**
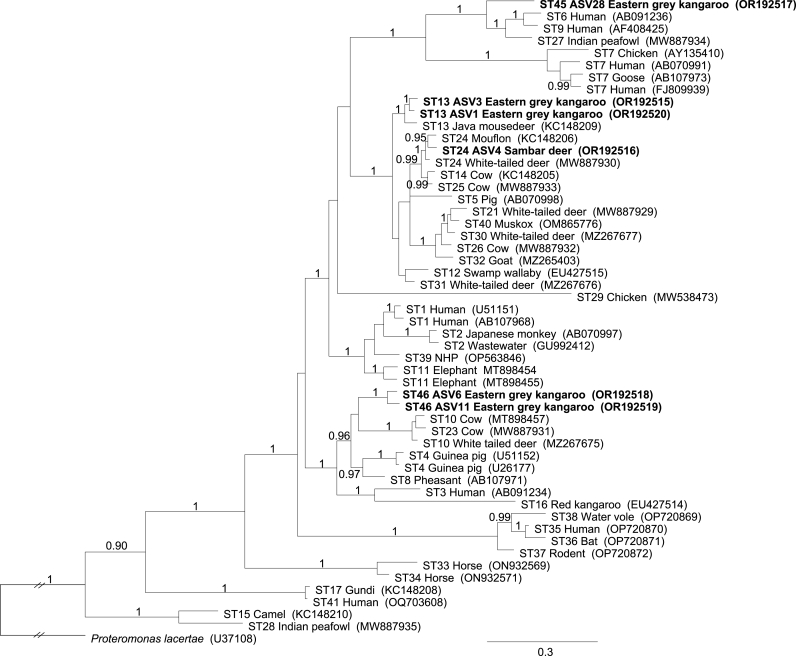
Phylogenetic analysis of *SSU*-rRNA sequence data (aligned over 2035 positions) to infer the relationships of recognised *Blastocystis* subtypes (STs) as well as new STs discovered in the present study. The tree was constructed using Bayesian Inference method (MrBayes) and used *Proteromonas lacertae* as an outgroup. Posterior probabilities less than 0.95% are not displayed. The two novel subtypes and additional ST13 and ST24 sequences are indicated in bold. After the present analysis was completed, Santín et al. (2023) reported a subdivision of “ST10” into four STs (i.e. ST10, ST42, ST43 and ST44).

DADA2 analysis identified 208 ASVs. After excluding ASVs with fewer than 100 sequence reads and chimeric sequences, 55 distinct ASVs were confirmed to represent all 356 test samples (cf. Supplementary file 3). These ASVs represented seven recognised STs (ST10, ST13, ST14, ST21, ST23, ST24 and ST25) and two new STs (ST45 and ST46) (Table 1). The identity of selected STs (including ST13 and ST24) and the novelty of ST45 and ST46 were verified via an extended analysis of *SSU*-*rRNA* (1717 to 1765 bp in length). ST45 was most similar (92.9%) in sequence to ST27 from an Indian peafowl (GenBank accession: MW887934; 100% coverage), and ST46 was most similar (93.0%) in sequence to ST10 from a camel (GenBank accession no. KC148207; 99% coverage). Neither ST45 nor ST46 clustered with any of the 37 currently-recognised STs in a phylogenetic analysis of *SSU*-*rRNA* sequence data, whereas all recognised STs clustered as expected (Fig. 3).

### 3.3 Prevalence of Blastocystis STs in the animal species

In marsupials, ST13 was prominent – present in 93.3% of kangaroos and wallabies – but was detected in only one sambar deer sample (Table 1), whereas ST10, ST14 and ST24 were identified in small numbers of samples from these marsupials (Table 1).

In red deer, ST24 was prominent – present in all samples – and ST21, ST14, ST10, ST23 and ST25 were recorded in 90%, 80%, 60%, 40% and 30% of samples, respectively (Table 1). In fallow deer, ST24, ST14, ST10, ST21, ST25 and ST23 were recorded in 100%, 96%, 88%, 88%, 76% and 72% of samples, respectively (Table 1), whereas ST21 was present in 94.7% of sambar deer samples, and ST24, ST10, ST25, ST14 and ST23 in 90.9%, 77.1%, 72.7%, 70.5% and 68.2% of these samples, respectively (Table 1). Although ST13 was detected in a sambar deer sample (0.8%) (Table 1), its presence might relate to pseudo-parasitism (cf. Koehler et al., 2016).

In other animals, ST13 and ST46 were both detected in one of two emu samples, and ST14, ST21, ST23, ST24 and ST25 in another sample. Both ST13 and ST46 were also recorded in one feral dog sample (Table 1).

Of the two novel STs identified, ST46 was identified in 93.9% of kangaroos and 33.3% of wallabies (Table 1), and ST45 in two kangaroo samples (1.1%) (Table 1).

### 3.4 Presence of multiple Blastocystis STs in most animal species

**Fig. 4:**
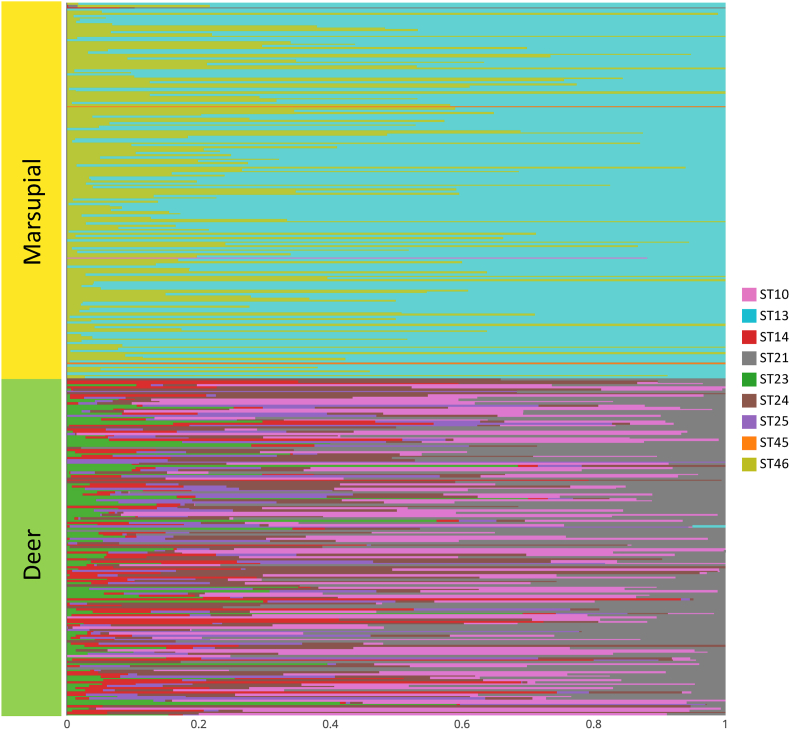
Relative abundance of *Blastocystis* subtypes (STs) in marsupial and deer species. Marsupials are represented by eastern grey kangaroos and wallabies; deer are represented by red, fallow and sambar deer. (For interpretation of the references to color in this figure legend, the reader is referred to the Web version of this article.)

Multiple STs were recorded in eastern grey kangaroo, swamp wallaby, red deer, fallow deer, sambar deer, canids and emu, but not in European rabbits or common wombats (Table 3, Fig. 4). Most eastern grey kangaroos (89.4%) harboured two to four STs, and 50.0 % of swamp wallabies had two STs (Table 3, Supplementary file 4). Of all eastern grey kangaroos with multiple STs, most (88.3%) had both ST13 and ST46 (Table 3). Interestingly, ST45 alone was present only in two kangaroo samples (see Fig. 4). Wallaby samples contained either a combination of ST13 and ST46 (33.3%), or of ST10 and ST13 (16.7%).

**Table 3:** Subtype combinations of *Blastocystis* in distinct animal species from Melbourne's water catchments identified using NGS-based sequencing-phylogenetic analysis (see section 2 for details on methods).

Species	No. of subtypes present in mixed infection	Subtype combination	No. of samples observed with the subtype combination	Percentage of samples with mixed infection out of total samples tested (%)
Eastern grey kangaroo	2	ST13/ST46	159	88.3
	3	ST14/ST21/ST24	1	0.6
	4	ST13/ST14/ST21/ST46	1	0.6
		**Total**	**161**	**89.4**
Swamp wallaby	2	ST13/ST46	2	33.3
		ST10/ST13	1	16.7
		**Total**	**3**	**50.0**
Red deer	2	ST21/ST24	1	10.0
	3	ST14/ST21/ST24	2	20.0
	4	ST10/ST14/ST21/ST24	2	20.0
	5	ST10/ST14/ST21/ST23/ST24	1	10.0
	6	ST10/ST14/ST21/ST23/ST24/ST25	3	30.0
		**Total**	**9**	**90.0**
Fallow deer	3	ST10/ST14/ST24	1	4.0
		ST14/ST21/ST24	2	8.0
		ST14/ST23/ST24	1	4.0
	4	ST10/ST21/ST24/ST25	1	4.0
	5	ST10/ST14/ST21/ST23/ST24	2	8.0
		ST10/ST14/ST23/ST24/ST25	1	4.0
		ST10/ST14/ST21/ST24/ST25	3	12.0
	6	ST10/ST14/ST21/ST23/ST24/ST25	14	56.0
		**Total**	**25**	**100.0**
Sambar deer	2	ST10/ST21	2	1.5
		ST14/ST24	2	1.5
		ST21/ST23	1	0.8
		ST21/ST24	6	4.5
		ST21/ST25	1	0.8
	3	ST10/ST21/ST23	1	0.8
		ST10/ST21/ST24	1	0.8
		ST14/ST21/ST24	6	4.5
		ST21/ST23/ST24	1	0.8
		ST21/ST24/ST25	1	0.8
	4	ST10/ST14/ST21/ST24	4	3.0
		ST10/ST13/ST14/ST25	1	0.8
		ST10/ST14/ST23/ST25	1	0.8
		ST10/ST21/ST23/ST24	2	1.5
		ST10/ST21/ST24/ST25	3	2.3
		ST10/ST23/ST24/ST25	2	1.5
		ST14/ST21/ST24/ST25	1	0.8
		ST14/ST23/ST24/ST25	1	0.8
		ST21/ST23/ST24/ST25	1	0.8
	5	ST10/ST14/ST21/ST23/ST24	7	5.3
		ST10/ST14/ST21/ST23/ST25	1	0.8
		ST10/ST14/ST21/ST24/ST25	12	9.1
		ST10/ST21/ST23/ST24/ST25	16	12.1
		ST14/ST21/ST23/ST24/ST25	5	3.8
	6	ST10/ST14/ST21/ST23/ST24/ST25	50	37.9
		**Total**	**129**	**97.7**
Emu	2	ST13/ST46	1	50.0
	5	ST14/ST21/ST23/ST24/ST25	1	50.0
		**Total**	**2**	**100.0**
Canid	2	ST13/ST46	1	100.0
		**Total**	**1**	**100.0**

Both red deer (90.0%) and sambar deer (97.0 %) harboured two to six different STs, and all fallow deer samples (100.0%) contained either three, four, five or six ST combinations (Table 3, Supplementary file 4). Most ST combinations (n = 24) were recorded in sambar deer, including mixes of two to six STs (Table 3), with the ST10/ST14/ST21/ST23/ST24/ST25 combination being commonest (37.9%; Table 3). In red and fallow deer, the commonest ST combinations were ST10/ST14/ST21/ST23/ST24/ST25 (30.0%) and ST10/ST14/ST21/ST23/ST24/ST25 (56.0%), respectively. In emu, the sole combinations were ST13/ST46 or ST14/ST21/ST23/ST24/ST25, whereas only one combination (ST13/ST46) was recorded in one feral dog sample (Table 3).

### 3.5 Seasonality and Blastocystis occurrence

According to the binary logistic model, season was shown to be strongly related with the occurrence (presence/absence) of *Blastocystis* in macropods (i.e., kangaroo and wallaby) (*p* < 0.05, **ᵡ**^2^ of 6.99*10^−4^), whereas, for deer, *Blastocystis* occurrence was not significantly linked to season (*p* > 0.05, **ᵡ**^2^ of 0.25) (Table 4). Furthermore, the odds of *Blastocystis* occurrence in macropods in summer were 1.88 times (88%) higher than that of autumn.

**Table 4:** The effect of season on the occurrence of *Blastocystis* in macropods and deer from Melbourne's water catchments. The results of binary logistic regression analysis showing the association of season with occurrence of *Blastocystis* in macropods (kangaroo and wallaby) and deer (red, fallow and sambar deer).

Host/Season	No. of samples tested	No. of positives	Prevalence (%)	Estimate	Standard error	Z value	*P* value	OR (95% CI)	ᵡ^2^
**Macropods**									
Autumn	173	35	20.23	Reference					
Spring	187	35	18.72	0.01	0.24	0.06	0.95	1.01 (0.63–1.64)	6.99 × 10^−4^***
Summer	232	106	45.69	0.63	0.22	2.85	0.004**	1.88 (1.22–2.92)	
Winter	244	45	18.44	−0.13	0.23	−0.55	0.58	0.88 (0.56–1.39)	
***Deer***									
Autumn	146	52	35.62	Reference					
Spring	130	44	33.85	−1.83 × 10^−16^	2.46 × 10^−1^	0.00	1.00	1.00 (0.62–1.62)	0.25
Summer	110	22	20.00	−4.80 × 10^−1^	2.64 × 10^−1^	−1.82	0.07	0.62 (0.37–1.04)	
Winter	152	49	32.24	−1.48 × 10^−1^	2.35 × 10^−1^	−0.63	0.53	0.86 (0.54–1.37)	

**Note:** OR = Odds Ratio, ᵡ^2^ = Chi-square, ***P < 0, **P < 0.001, *P < 0.01.

## 4 Discussion

The present study investigated *Blastocystis* in a range of Australian wildlife, utilising a considerable number of faecal samples (1,658) collected from water catchment areas in Victoria, Australia, over a period from 2009 to 2022. We identified seven currently-recognised STs in marsupial, deer and/or canines, and two novel STs predominantly in kangaroos. Multiple STs (two to six) were recorded primarily in both macropods and deer. Of these, only ST10, ST14 and ST23 have been recently recorded in humans (Khaled et al., 2020; Jinatham et al., 2021).

ST13 predominated in macropodid marsupials (i.e., kangaroo and wallaby) in the present study (see Table 1) and met the current guide for the designation of a novel ST (cf. Stensvold and Clark, 2020): i.e. >80% of the sequence of *SSU-rRNA* gene region was obtained, and (2) the sequence variability/difference was ≥4%. We propose that ST13 is primarily a marsupial ST. Originally, this ST was identified in quokka (*Setonix brachyurus*) from the Perth Zoo based on a partial *SSU-rRNA* sequence (1015 bp) (Parkar et al., 2010), and also in a Java mousedeer (*Tragulus javanicus*) from Paignton Zoo, UK, based on the entire *SSU-rRNA* gene (Alfellani et al., 2013). Subsequently, ST13 was recorded in a related Java mousedeer in France within the context of a shared zoo program (Cian et al., 2017). This ST has also been found in a “white kangaroo” from a breeding Chinese breeding facility (K. Zhang et al., 2021), which is most likely a red kangaroo; *Macropus rufus* (personal communication with Dr Zhijun Zhong, 27 September 2017). Clearly, caution is warranted in making conclusions about an affiliation with a particular animal species in a captive environment, such as in parks, zoos or breeding/production facilities, due to cross transmission events of *Blastocystis* among species. There are also reports of ST13 in cervids in China, South Korea and UK (Alfellani et al., 2013; Betts et al., 2018; Wang et al., 2018; Kim et al., 2020; Li et al., 2020; Chen et al., 2021, 2022) and from primates in Bangladesh, China and Poland (Li et al., 2019; Rudzińska et al., 2021; Geng, 2021- unpublished; Li et al., 2022 - unpublished), but none of these studies has provided sequence for >80% of the *SSU-rRNA* gene. Other key STs recorded in kangaroos and wallabies in previous studies include ST12, in the western grey kangaroo and northern swamp wallaby from zoos (Parkar et al., 2010) and ST16, in red kangaroos from a zoo (Yoshikawa, 2011 - unpublished) (cf. Supplementary file 5).

Although the first record of *Blastocystis* ST4 in an eastern grey kangaroo was from Taronga Zoo, New South Wales, Australia (Roberts et al., 2013), interestingly, this ST was not identified in any of the samples tested here. Moreover, prior to this study, several other studies of wild and/or captive marsupials, including the western grey kangaroo, red kangaroo, western quoll, opossum, wallaby, eastern wallaroo, southern brown bandicoot and brushtail possum, reported ST1 to ST5, ST7, ST8, ST10, ST12, ST13 and ST16 (see Supplementary file 5 for references). However, *Blastocystis* STs detected in captive animals in zoos, wildlife parks or farms may not reflect those occurring naturally in animals in the wild, because they could readily be acquired *via* cross transmission from humans and/or a range of species in high density environment/situation (Parkar et al., 2010).

It is important to note that ST46, the prominent, new ST identified in macropods, co-occurred with ST13 in most (88.3%) samples from eastern grey kangaroos, whereas the other novel ST, ST45, was solely detected in only two samples (1.1%) from this macropod species. Therefore, the substantial number of samples tested in this study was crucial in identifying these rare STs, which, otherwise, would have been overlooked.

Faecal samples from wombats were collected from the same locations as eastern grey kangaroos and swamp wallabies. As these species have partially overlapping diets (Davis et al., 2008), we suspect that genetic, physiological and/or or immunological barriers in the common wombat might account for the apparent absence of *Blastocystis* in this host species in the present study; further studies of the different species of wombat should help elaborate the results observed herein. Previously, one case of *Blastocystis* ST1 was recorded in a southern hairy-nosed wombat (*Lasiorhinus latifrons*) in the Perth Zoo (Parkar et al., 2010), but no *Blastocystis* was detected by molecular means in samples from three southern hairy-nosed wombats and two common wombats from the Taronga Zoo in Australia (Roberts et al., 2013).

Most reports of *Blastocystis* from deer are based on studies of limited sample sizes (<5) (Stensvold et al., 2009a; Hublin et al., 2021; see Supplementary file 5), with ST10 and ST14 being the most reported in cervids as well as other ruminants (Hublin et al., 2021). Melbourne's water catchments are inhabited by sambar, red and fallow deer, which were introduced to Australia in the late 1800s from parts of Europe and Asia (cf. Koehler et al., 2020). Although some studies have tested faecal samples from these three species of deer for *Blastocystis*, most animal were captive (e.g., in zoos or on farms) (see Supplementary file 5 for references). An exception is a study conducted in the Sichuan Wolong National Natural Reserve in Southwest China, in which 8 (of 39 tested) wild sambar deer harboured *Blastocystis* ST13 and/or ST14 (Chen et al., 2021). However, the validity of ST13 is in question because <80% of the *SSU-rRNA* gene was sequenced (cf. Stensvold and Clark, 2020). Red deer have been reported to harbour ST4 (Roberts et al., 2013; Betts et al., 2018) and ST10 (Betts et al., 2018) in the UK, and ST1 in Poland (Kaczmarek et al., 2021). Fallow deer have been recorded to harbour ST5 in Italy (Gabrielli et al., 2021) and ST10 in China and Mauritius (Alfellani et al., 2013; Zhao et al., 2017).

The prevalence of *Blastocystis* in studies performed using relatively large numbers of faecal samples from wild deer species and PCR-based sequencing, ranges from 29% (83/286) (Zhang et al., 2022) to 88.8% (71/80) (Maloney et al., 2021), compared with 45.5% (246/540) in the present study. However, previous studies are not as comprehensive as the current study and are not comparable for a variety of reasons, primarily because NGS was not performed and mixed infections were not identified previously, except in the study by Maloney et al. (2021). Regardless, it is still useful to compare dominant STs of *Blastocystis* among studies. For instance, Kim et al. (2020) examined 125 samples (40.8% positive) from wild Korean water deer (*Hydroptes inermis argyropus*) and recorded ST4 and ST14, and did not detect a seasonal difference in occurrence between spring and summer. Zhang et al. (2022) and Ni et al. (2023) both examined Père David's deer, which were extirpated from China in the 19th century, and later re-populated to nature reserves in Beijing and Shishou from a small population of captive deer surviving in the UK (Y. Zhang et al., 2021). The study of Père David's deer performed in Beijing (*n* = 286 samples) revealed a *Blastocystis* prevalence of 29%, and identified ST10, ST14 and ST21 over a four-year period (Zhang et al., 2022), whereas a study in Shishou (*n* = 128) established a *Blastocystis* prevalence of 56.3%, identifying ST10, ST21, ST23, ST25 and ST26 over a week in the summer of 2018 (Ni et al., 2023). Unfortunately, in a study of 132 wild Yezo sika deer (*Cervus nippon yesoensis*), revealing a *Blastocystis* prevalence of 45.5%) only eight of 60 PCR products were sequenced and exclusively revealed ST14 (Shirozu et al., 2021). Interestingly, the dominant STs observed in these previous studies were also recorded in the present study (i.e., ST10, ST14, ST21, ST23 and ST25).

Maloney et al. (2021) is the only other study that has used NGS of part of the *SSU-rRNA* gene (500 bp) to genetically characterise *Blastocystis* in deer, making it useful for comparative purposes. This study was conducted using faecal samples supplied from a managed hunt of white-tailed deer (*Odocoileus virginianus*) from Maryland, USA, collected over two consecutive hunting seasons (late autumn). All six STs recorded (i.e., ST10, ST14, ST21, ST23, ST24 and ST25) in the present study were also found by Maloney et al. (2021), but these STs were more consistently distributed herein among the individual samples (prevalence: ≥65%) compared with a sporadic distribution of STs in the study by Maloney et al. (2021) (Supplementary file 6). The additional STs found in the latter study included ST1, ST3 and ST4, three human-infective STs. However, these three STs were detected at low prevalence (<5%), which might be reflective of the residential areas in which the deer were hunted, compared with the drinking water catchments in which the deer samples were collected in the present study. In these catchment areas, there is likely no human contact and the deer tend to stay behind the relatively protected gated catchments. Two novel STs, designated ST30 and ST31, were also found in the study by Maloney et al. (2021), which may be a species- or location-specific ST.

Comprehensive epidemiological analysis to determine the effects of environmental factors and/or demographic factors of hosts on the occurrence and/or diversity of *Blastocystis* is lacking in most of the previous studies, except for Kim et al. (2020). The findings of the present study indicate a significant effect of season on *Blastocystis* occurrence in macropods but not in deer. Future, comprehensive epidemiological analyses should provide better insight into seasonal effects.

The worldwide prevalence of *Blastocystis* ST3, the most frequent ST in dogs, is 7%, according to a meta-analysis of *Blastocystis* in dogs (Shams et al., 2022). In the present study, we tested 94 samples from canids (feral dogs, dingo or foxes) and found one test-positive sample (∼1.0%) - a co-occurrence of *Blastocystis* ST13 and ST46, which is most likely the result of a canine eating a macropod. A mob of emus that were introduced to Cardinia catchment in the late 1980's was also sampled over a 12-year period. Of the 94 samples tested here, only two (2.1%) - a co-occurrence of *Blastocystis* ST14/ST21/ST23/ST24/ST25 in one sample and another sample with ST13/ST46 - were positive for *Blastocystis* by PCR. The emus in this catchment have been known to acquire other parasites, most likely through coprophagy (pseudo-parasitism), as was the case for *Cryptosporidium canis* in an emu (Koehler et al., 2016). The fact that the emus had *Blastocystis* STs found in both macropods and deer might support this proposal.

Similar to the wombats, none of the rabbits tested in this study were positive for *Blastocystis*. This appears to be the first investigation of feral rabbits for *Blastocystis*; all previous studies explored captive rabbits (Wang et al., 2018; Abuodeh et al., 2019; Li et al., 2020; Su et al., 2022). The STs observed in these previous studies include, ST1, ST3, ST4, ST7 and ST14 (Wang et al., 2018; Abuodeh et al., 2019; Li et al., 2020; Su et al., 2022). Some other studies did not detect *Blastocystis* in rabbits, but samples sizes were too low (*n* = 1-4; Alfellani et al., 2013; Roberts et al., 2013; Higuera et al., 2021; Rudzińska et al., 2022) to be conclusive. Nonetheless, a *Blastocystis* prevalence of 0.97% was observed in a study with the largest sample size of rabbits (*n* = 616; Li et al., 2020), and a study by Su et al. (2022) reported the highest prevalence (15%) of *Blastocystis* in rabbits and marked ST diversity (i.e., ST1, ST3 and ST7).

Findings from the present and previous studies (Calero-Bernal et al., 2020; Maloney et al., 2019, 2020, 2021, 2023; Russini et al., 2020; Abarca et al., 2021; Higuera et al., 2021; Baek et al., 2022) which have used NGS of *SSU-rRNA* gene regions (320-500 bp) to genetically characterise *Blastocystis* show that PCR alone is inadequate to detect ST diversity within individual samples. In the present study, 97% of the samples from deer contained multiple STs, with most (71%) having five to six STs; this finding is similar to that of Maloney et al. (2021) who found that 90.1% (64/71) of white-tailed deer samples contained multiple STs (see Table 3, Supplementary file 4). Furthermore, in the current study, 50 of the 132 samples (37.9%) from sambar deer contained all six STs identified in this species (ST10/ST14/ST21/ST23/ST24/ST25). Only small numbers of samples from deer contained single STs in the present study (2.4%; ST21 and 25 in two and one sambar deer sample/s, respectively, and ST24 in one red deer sample). In the study by Maloney et al. (2021) only 9.9% (7/71) of white-tailed deer were shedding one ST. By comparison, 9 (4.8%) of the 233 macropod samples tested here contained a single *Blastocystis* ST (Table 5), lending support to the proposal that conventional PCR is not suited for the accurate identification of multiple STs in individual samples.

**Table 5:** The occurrence of single subtype infections in tested host species from Melbourne's water catchments.

	ST10	ST13	ST14	ST21	ST23	ST24	ST25	ST45	ST46
Eastern grey kangaroo	0	8	0	0	0	0	0	2	9
Swamp wallaby	0	3	0	0	0	0	0	0	0
Red deer	0	0	0	0	0	1	0	0	0
Fallow deer	0	0	0	0	0	0	0	0	0
Sambar deer	0	0	0	2	0	0	1	0	0
Unknown deer	0	0	0	0	0	0	0	0	0
Emu	0	0	0	0	0	0	0	0	0
Canid	0	0	0	0	0	0	0	0	0
Common wombat	0	0	0	0	0	0	0	0	0
European rabbit	0	0	0	0	0	0	0	0	0
**Total**	**0**	**11**	**0**	**2**	**0**	**1**	**1**	**2**	**9**

For the most part, STs recorded in this study were specific to either macropods or deer. On a few occasions, STs (i.e. ST14, ST21 and ST24) known to occur in deer (Maloney et al., 2021) were observed in macropod samples, and ST13 (typically found in macropods) in a deer sample. Given this very occasional occurrence of an apparently unusual ST, we suspect that such records might relate to contamination (during sample collection) or pseudo-parasitism, as deer and macropod faeces co-occur in water catchment areas. The sequencing results obtained here show that was no evidence of cross-contamination between/among wells, as there was a clear and consistent distinctiveness in STs between macropod and deer samples (located in a randomized manner to wells).

Clark et al. (2013) first suggested that distinct STs should differ by ≥ 4% over ≥80% of the entire *SSU-rRNA* gene region. Over time, it is expected that significantly more sequence data will become available for “novel” STs (in GenBank), the precise phylogenetic assignment of STs will likely become challenging using the 4% cut-off. This challenge has been demonstrated here for ST13. Given the challenges in assigning species, genotypes and STs, based on a single gene alone, it would be advantageous to employ multiple (preferably many) markers to assign ST status in the future (cf. Clark et al., 2013).

## 5 Conclusion

The present study reports, for the first time, the occurrence and diversity of *Blastocystis* in Australian wildlife, including marsupials, deer, emus, rabbits and canids, using relatively large sample sizes. This study reveals the patterns of occurrence/co-occurrence of different STs in different hosts, and suggests that some STs are cross-transmissible among different host species, and that the occurrence of human-infective STs in wildlife presents a potential risk for the contamination of water catchments. In the future, extensive analyses of a range of other wild animal hosts from different states would provide a more complete picture of the *Blastocystis* prevalence, ST diversity and determinants for ST occurrence in wildlife in Australia, and the potential of wildlife being reservoirs for zoonotic transmission.

## Funding

This study was supported by 10.13039/501100004170Melbourne Water Corporation through the 10.13039/501100000923Australian Research Council (ARC) Linkage Project grant LP160101299. Funding bodies played no role in the design of the study, collection, analysis or interpretation of data, or in the writing of the manuscript.

## Ethical statement

The author confirms that the ethical policies of the journal, as noted in the journal's author guidelines, have been adhered to. No ethical approval was required for analysis of sample material.

## Credit author statement

Conceptualisation, A.V.K. and R.B.G; methodology, A.V.K., H.M.P.D.H, R.S.H, S.W. and R.B.G; formal analysis, A.V.K., H.M.P.D.H, R.S.H and R.B.G; resources, R.S.H., S.W. and R.B.G; data curation, A.V.K., R.S.H and S.W.; writing—original draft preparation, A.V.K., H.M.P.D.H. and R.B.G; writing—review and editing, A.V.K., H.M.P.D.H. and R.B.G; project administration, A.V.K. and R.B.G; funding acquisition, A.V.K. and R.B.G. All authors have read and agreed to the published version of the manuscript.

## Data availability

All the data used in the present study are reported in the manuscript and in the relative supplementary information. The obtained sequences are available in GenBank (OR192515 - OR192520). Raw data provided in BioProject PRJNA987751.

## Declaration of competing interest

The authors declare the following financial interests/personal relationships which may be considered as potential competing interests: Anson Koehler reports financial support was provided by the 10.13039/501100000923Australian Research Council.

## References

[bib1] Abarca N., Santín M., Ortega S., Maloney J.G., George N.S., Molokin A., Cardona G.A., Dashti A., Köster P.C., Bailo B., Hernández-de-Mingo M. (2021). Molecular detection and characterization of *Blastocystis* sp. and *Enterocytozoon bieneusi* in cattle in Northern Spain. Vet. Sci..

[bib2] Abuodeh R., Ezzedine S., Madkour M., Stensvold C.R., Samie A., Nasrallah G., AlAbsi E., ElBakri A. (2019). Molecular subtyping of *Blastocystis* from diverse animals in the United Arab Emirates. Protist.

[bib3] Alfellani M.A., Taner-Mulla D., Jacob A.S., Imeede C.A., Yoshikawa H., Stensvold C.R., Clark C.G. (2013). Genetic diversity of *Blastocystis* in livestock and zoo animals. Protist.

[bib4] Andersen L.O.B., Stensvold C.R. (2016). *Blastocystis* in health and disease: are we moving from a clinical to a public health perspective?. J. Clin. Microbiol..

[bib5] Aubrey B.J., Kelly G.L., Kueh A.J., Brennan M.S., O'Connor L., Milla L., Wilcox S., Tai L., Strasser A., Herold M.J. (2015). An inducible lentiviral guide RNA platform enables the identification of tumor-essential genes and tumor promoting mutations in vivo. Cell Rep..

[bib6] Baek S., Maloney J.G., Molokin A., George N.S., Cortés Vecino J.A., Santín M. (2022). Diversity of *Blastocystis* subtypes in horses in Colombia and identification of two new subtypes. Microorganisms.

[bib7] Betts E.L., Gentekaki E., Thomasz A., Breakell V., Carpenter A.I., Tsaousis A.D. (2018). Genetic diversity of *Blastocystis* in non-primate animals. Parasitology.

[bib8] Calero‐Bernal R., Santín M., Maloney J.G., Martín‐Pérez M., Habela M.A., Fernández‐García J.L., Figueiredo A., Nájera F., Palacios M.J., Mateo M., Balseiro A. (2020). *Blastocystis* sp. subtype diversity in wild carnivore species from Spain. J. Eukaryot. Microbiol..

[bib9] Callahan B.J., McMurdie P.J., Rosen M.J., Han A.W., Johnson A.J.A., Holmes S.P. (2016). DADA2: high-resolution sample inference from Illumina amplicon data. Nat. Methods.

[bib10] Cian A., El Safadi D., Osman M., Moriniere R., Gantois N., Benamrouz-Vanneste S., Delgado-Viscogliosi P., Guyot K., Li L.L., Monchy S., Noël C. (2017). Molecular epidemiology of *Blastocystis* sp. in various animal groups from two French zoos and evaluation of potential zoonotic risk. PLoS One.

[bib11] Chen S., Meng W., Shi X., Chai Y., Zhou Z., Liu H., Zhong Z., Fu H., Cao S., Ma X., Shen L. (2022). Occurrence, genetic diversity and zoonotic potential of *Blastocystis* sp. in forest musk deer (*Moschus berezovskii*) in Southwest China. Parasite.

[bib12] Chen S., Meng W., Zhou Z., Deng L., Shi X., Chai Y., Liu H., Cheng Y., Zhong Z., Fu H., Shen L. (2021). Genetic characterization and zoonotic potential of *Blastocystis* from wild animals in sichuan Wolong national natural reserve, southwest China. Parasite.

[bib13] Clark C.G., van der Giezen M., Alfellani M.A., Stensvold C.R. (2013). Recent developments in *Blastocystis* research. Adv. Parasitol..

[bib14] Crossley B.M., Bai J., Glaser A., Maes R., Porter E., Killian M.L., Clement T., Toohey-Kurth K. (2020). Guidelines for Sanger sequencing and molecular assay monitoring. J. Vet. Diagn. Invest..

[bib15] Davis N.E., Coulson G., Forsyth D.M. (2008). Diets of native and introduced mammalian herbivores in shrub-encroached grassy woodland, south-eastern Australia. Wildl. Res..

[bib16] Deng L., Wojciech L., Gascoigne N.R., Peng G., Tan K.S. (2021). New insights into the interactions between *Blastocystis*, the gut microbiota, and host immunity. PLoS Pathog..

[bib17] Edgar R.C. (2004). MUSCLE: multiple sequence alignment with high accuracy and high throughput. Nucleic Acids Res..

[bib18] Gabrielli S., Palomba M., Furzi F., Brianti E., Gaglio G., Napoli E., Rinaldi L., Alburqueque R.A., Mattiucci S. (2021). Molecular subtyping of *Blastocystis* sp. isolated from farmed animals in southern Italy. Microorganisms.

[bib19] Greige S., El Safadi D., Becu N., Gantois N., Pereira B., Chabé M., Benamrouz-Vanneste S., Certad G., El Hage R., Chemaly M., Hamze M., Viscogliosi E. (2018). Prevalence and subtype distribution of *Blastocystis* sp. isolates from poultry in Lebanon and evidence of zoonotic potential. Parasites Vectors.

[bib20] Higuera A., Herrera G., Jimenez P., Garcia-Corredor D., Pulido-Medellin M., Bulla-Castañeda D.M., Pinilla J.C., Moreno-Pérez D.A., Maloney J.G., Santín M., Ramírez J.D. (2021). Identification of multiple *Blastocystis* subtypes in domestic animals from Colombia using amplicon-based next generation sequencing. Front. Vet. Sci..

[bib21] Hublin J.S., Maloney J.G., Santín M. (2021). *Blastocystis* in domesticated and wild mammals and birds. Res. Vet. Sci..

[bib22] Jinatham V., Maxamhud S., Popluechai S., Tsaousis A.D., Gentekaki E. (2021). *Blastocystis* One Health approach in a rural community of Northern Thailand: prevalence, subtypes and novel transmission routes. Front. Microbiol..

[bib23] Kaczmarek A., Sobociński W., Wesołowska M., Gołąb E., Kołodziej-Sobocińska M., Sałamatin R. (2021). *Blastocystis* occurrence and subtype diversity in wild European terrestrial mammals - the case of Białowieża Primeval Forest (NE Poland). Int. J. Parasitol. Parasit. Wildlife..

[bib24] Khaled S., Gantois N., Ly A.T., Senghor S., Even G., Dautel E., Dejager R., Sawant M., Baydoun M., Benamrouz-Vanneste S., Chabé M. (2020). Prevalence and subtype distribution of *Blastocystis* sp. in Senegalese school children. Microorganisms.

[bib25] Kim K.T., Noh G., Lee H., Kim S.H., Jeong H., Kim Y., Jheong W.H., Oem J.K., Kim T.H., Kwon O.D., Kwak D. (2020). Genetic diversity and zoonotic potential of *Blastocystis* in Korean water deer, *Hydropotes inermis argyropus*. Pathogens.

[bib26] Koehler A.V., Haydon S.R., Jex A.R., Gasser R.B. (2016). *Cryptosporidium* and *Giardia* taxa in faecal samples from animals in catchments supplying the city of Melbourne with drinking water (2011 to 2015). Parasites Vectors.

[bib27] Koehler A.V., Zhang Y., Wang T., Haydon S.R., Gasser R.B. (2020). Multiplex PCRs for the specific identification of marsupial and deer species from faecal samples as a basis for non-invasive epidemiological studies of parasites. Parasites Vectors.

[bib28] Lee M.D. (2019). Happy Belly Bioinformatics: an open-source resource dedicated to helping biologists utilize bioinformatics. J. Open Source Educ..

[bib29] Li J., Karim M.R., Li D., Sumon S.M.R., Siddiki S.F., Rume F.I., Sun R., Jia Y., Zhang L. (2019). Molecular characterization of *Blastocystis* sp. in captive wildlife in Bangladesh National Zoo: non-human primates with high prevalence and zoonotic significance. Int. J. Parasitol. Parasit. Wildlife..

[bib30] Li T.S., Zou Y., Ma Y.T., Ma Y.Y., Chen H., Liang X.X., Cong W., Sun X.L., Zhu X.Q. (2020). Molecular characterization of *eimeria* spp. and *Blastocystis* in rabbits in Shandong Province, China. Parasitol. Res..

[bib31] Maddison W.P., Maddison D.R. (2015). Mesquite: a modular system for evolutionary analysis v.3.04. http://mesquiteproject.org.

[bib32] Maloney J.G., Jang Y., Molokin A., George N.S., Santín M. (2021). Wide genetic diversity of *Blastocystis* in white-tailed deer (*Odocoileus virginianus*) from Maryland, USA. Microorganisms.

[bib33] Maloney J.G., Molokin A., Santín M. (2019). Next generation amplicon sequencing improves detection of *Blastocystis* mixed subtype infections. Infect. Genet. Evol..

[bib34] Maloney J.G., Molokin A., Santín M. (2020). Use of Oxford Nanopore MinION to generate full-length sequences of the *Blastocystis* small subunit (SSU) rRNA gene. Parasites Vectors.

[bib35] Maloney J.G., Molokin A., Seguí R., Maravilla P., Martínez-Hernández F., Villalobos G., Tsaousis A.D., Gentekaki E., Muñoz-Antolí C., Klisiowicz D.R., Oishi C.Y. (2023). Identification and molecular characterization of four new *Blastocystis* subtypes designated ST35-ST38. Microorganisms.

[bib36] Maloney J.G., Santín M. (2021). Mind the gap: new full-length sequences of *Blastocystis* subtypes generated via Oxford Nanopore Minion sequencing allow for comparisons between full-length and partial sequences of the small subunit of the ribosomal RNA gene. Microorganisms.

[bib67] Martin M. (2011). Cutadapt removes adapter sequneces from high-throughput sequencing reads. EMBnet J.

[bib37] Minh B.Q., Schmidt H.A., Chernomor O., Schrempf D., Woodhams M.D., Von Haeseler A., Lanfear R. (2020). IQ-TREE 2: new models and efficient methods for phylogenetic inference in the genomic era. Mol. Biol. Evol..

[bib38] Murali A., Bhargava A., Wright E.S. (2018). IDTAXA: a novel approach for accurate taxonomic classification of microbiome sequences. Microbiome.

[bib39] Ni F., Yu F., Yang X., An Z., Ge Y., Liu X., Qi M. (2023). Identification and genetic characterization of *Blastocystis* subtypes in Père David's deer (*Elaphurus davidianus*) from Shishou, China. Vet. Res. Commun..

[bib40] Parkar U. (2016).

[bib41] Parkar U., Traub R.J., Vitali S., Elliot A., Levecke B., Robertson I., Geurden T., Steel J., Drake B., Thompson R.C.A. (2010). Molecular characterization of *Blastocystis* isolates from zoo animals and their animal-keepers. Vet. Parasitol..

[bib42] Roberts T., Stark D., Harkness J., Ellis J. (2013). Subtype distribution of *Blastocystis* isolates from a variety of animals from New South Wales, Australia. Vet. Parasitol..

[bib43] Ronquist F., Teslenko M., Van Der Mark P., Ayres D.L., Darling A., Höhna S., Larget B., Liu L., Suchard M.A., Huelsenbeck J.P. (2012). MrBayes 3.2: efficient Bayesian phylogenetic inference and model choice across a large model space. Syst. Biol..

[bib44] Rudzińska M., Kowalewska B., Kurpas M., Szostakowska B. (2022). Rare occurrence of *Blastocystis* in pet animals and their owners in the Pomeranian Voivodeship in Poland in the light of literature data. J. Clin. Med..

[bib45] Rudzińska M., Kowalewska B., Waleron M., Kalicki M., Sikorska K., Szostakowska B. (2021). Molecular characterization of *Blastocystis* from animals and their caregivers at the gdansk zoo (Poland) and the assessment of zoonotic transmission. Biology.

[bib46] Russini V., Di Filippo M.M., Fanelli R., Polidori M., Berrilli F., Di Cave D., Novelletto A., Calderini P. (2020). Characterization of prevalence and genetic subtypes of *Blastocystis* sp. in wild and domestic Suidae of central Italy aided by amplicon NGS. Vet. Parasitol. Reg. Stud. Rep..

[bib47] Santín M., Figueiredo A., Molokin A., George N.S., Köster P.C., Dashti A., González‐Barrio D., Carmena D., Maloney J.G. (2023). Division of *Blastocystis* ST10 into three new subtypes: ST42‐ST44. J. Eukaryot. Microbiol..

[bib48] Santín M., Gómez-Muñoz M.T., Solano-Aguilar G., Fayer R. (2011). Development of a new PCR protocol to detect and subtype *Blastocystis* spp. from humans and animals. Parasitol. Res..

[bib49] Scicluna S.M., Tawari B., Clark C.G. (2006). DNA barcoding of *Blastocystis*. Protist.

[bib50] Shams M., Shamsi L., Yousefi A., Sadrebazzaz A., Asghari A., Mohammadi-Ghalehbin B., Shahabi S., Hatam G. (2022). Current global status, subtype distribution and zoonotic significance of *Blastocystis* in dogs and cats: a systematic review and meta-analysis. Parasites Vectors.

[bib51] Shirozu T., Morishita Y.K., Koketsu M., Fukumoto S. (2021). Molecular detection of *Blastocystis* sp. subtype 14 in the Yezo sika deer (*Cervus nippon yesoensis*) in Hokkaido, Japan. Vet. Parasitol. Reg. Stud. Rep..

[bib52] Stensvold C.R., Alfellani M.A., Nørskov-Lauritsen S., Prip K., Victory E.L., Maddox C., Nielsen H.V., Clark C.G. (2009). Subtype distribution of *Blastocystis* isolates from synanthropic and zoo animals and identification of a new subtype. Int. J. Parasitol..

[bib53] Stensvold C.R., Clark C.G. (2016). Current status of *Blastocystis*: a personal view. Parasitol. Int..

[bib54] Stensvold C.R., Clark C.G. (2020). Pre-empting Pandora's box: *Blastocystis* subtypes revisited. Trends Parasitol..

[bib68] Stensvold C.R., Lebbad M., Victory E.L., Verweij J.J., Tannich E., Alfellani M., Legarraga P., Clark C.G. (2011). Increased sampling reveals novel lineages of Entamoeba: consequences of genetic diversity and host specificity for taxonomy and molecular detection. Protist.

[bib55] Stensvold C.R., Nielsen H.V., Mølbak K., Smith H.V. (2009). Pursuing the clinical significance of *Blastocystis*–diagnostic limitations. Trends Parasitol..

[bib56] Su C., Mei X., Feng X., Zhang F., Wang P., He B., Xu F., Yang Z., Tian X., Zhang Z., Li X. (2022). Prevalence and molecular subtyping of *Blastocystis* sp. in rabbits in Henan, Central China. Folia Parasitol..

[bib57] Tamura K., Stecher G., Kumar S. (2021). MEGA11: molecular evolutionary genetics analysis version 11. Mol. Biol. Evol..

[bib58] Triggs B. (2004).

[bib59] Wang J., Gong B., Liu X., Zhao W., Bu T., Zhang W., Liu A., Yang F. (2018). Distribution and genetic diversity of *Blastocystis* subtypes in various mammal and bird species in Northeastern China. Parasites Vectors.

[bib60] Wang W., Owen H., Traub R.J., Cuttell L., Inpankaew T., Bielefeldt-Ohmann H. (2014). Molecular epidemiology of *Blastocystis* in pigs and their in-contact humans in Southeast Queensland, Australia, and Cambodia. Vet. Parasitol..

[bib61] Yoshikawa H., Abe N., Iwasawa M., Kitano S., Nagano I., Wu Z., Takahashi Y. (2000). Genomic analysis of *Blastocystis hominis* strains isolated from two long-term health care facilities. J. Clin. Microbiol..

[bib62] Zhang K., Zheng S., Wang Y., Wang K., Wang Y., Gazizova A., Han K., Yu F., Chen Y., Zhang L. (2021). Occurrence and molecular characterization of *Cryptosporidium* spp., *Giardia duodenalis, Enterocytozoon bieneusi*, and *Blastocystis* sp. in captive wild animals in zoos in Henan, China. BMC Vet. Res..

[bib63] Zhang P., Zhang Q., Han S., Yuan G., Bai J., He H. (2022). Occurrence and genetic diversity of the zoonotic enteric protozoans and *Enterocytozoon bieneusi* in Père David's deer (*Elaphurus davidianus*) from Beijing, China. Pathogens.

[bib64] Zhang Y., Bai J., Zhu A., Chen R., Xue D., Zhong Z., Cheng Z. (2021). Reversing extinction in China's Père David's deer. Science.

[bib65] Zhao G.H., Hu X.F., Liu T.L., Hu R.S., Yu Z.Q., Yang W.B., Wu Y.L., Yu S.K., Song J.K. (2017). Molecular characterization of *Blastocystis* sp. in captive wild animals in Qinling Mountains. Parasitol. Res..

[bib66] Zhao Y., Federico A., Faits T., Manimaran S., Segrè D., Monti S., Johnson W.E. (2021). Animalcules: interactive microbiome analytics and visualization in R. Microbiome.

