# Supplementary material for: Marked genetic diversity within Blastocystis in Australian wildlife revealed using a next generation sequencing–phylogenetic approach
Source: Int J Parasitol Parasites Wildl. 2023 Dec 28;23:100902. doi: 10.1016/j.ijppaw.2023.100902 (PMC10827504; doi:10.1016/j.ijppaw.2023.100902)
Supplement: Multimedia component 2 [file mmc2.docx]

**Supplementary file 2.** Neighbor-joining tree constructed from *SSU* sequence data (180 bp) for *Blastocystis* sp., aligned using MAFFT. Sequences include the 55 ASVs and their top BLAST hits (with percentage identity following the ASV name). Major ASVs identified here are in bold. Subtype (ST) assignments are indicated (right). *Mis-assigned ST (from GenBank; unpublished data).
