# Supplementary figures and images for: Marked genetic diversity within Blastocystis in Australian wildlife revealed using a next generation sequencing–phylogenetic approach
Source: Int J Parasitol Parasites Wildl. 2023 Dec 28;23:100902. doi: 10.1016/j.ijppaw.2023.100902 (PMC10827504; doi:10.1016/j.ijppaw.2023.100902)

Supplementary file 4. Sequence read counts representing *Blastocystis* subtypes – sorted by year.

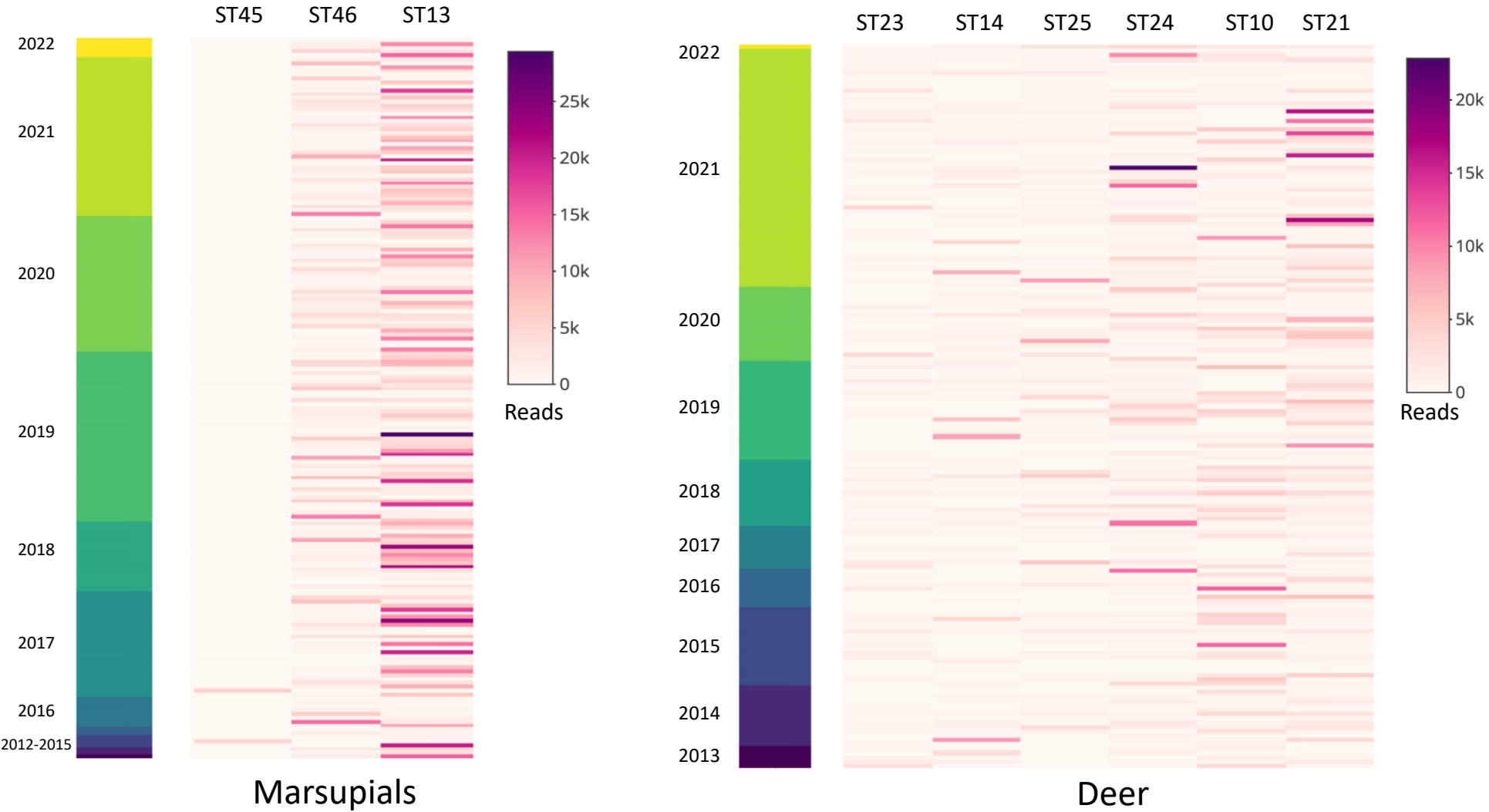

Supplement: Multimedia component 3 [file mmc3.pdf]
